# Supplementary material for: Hepatic IFNL4 Gene Activation in Hepatocellular Carcinoma Patients with Regard to Etiology
Source: Int J Mol Sci. 2021 Jul 21;22(15):7803. doi: 10.3390/ijms22157803 (PMC8345952; doi:10.3390/ijms22157803)
Supplement: Supplementary file 1 [file ijms-22-07803-s001.zip › ijms-1242824-supplementary.pdf]

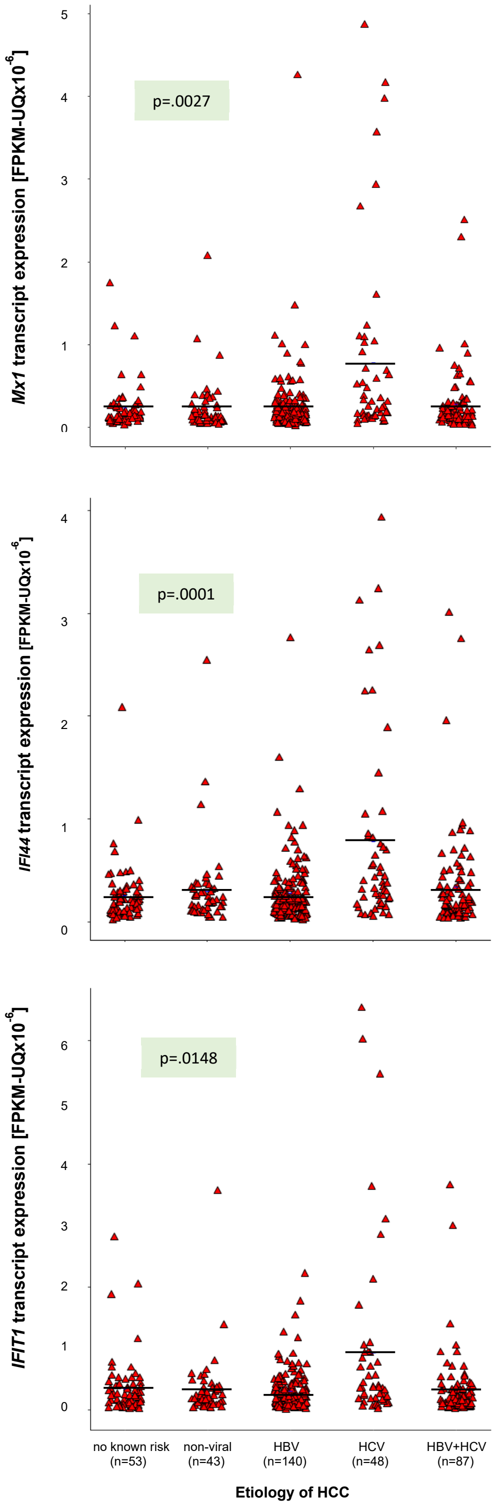

Figure S1: Interferon-stimulated gene (ISG) transcript expression in hepatocellular carcinoma (HCC) tissue samples with regard to etiology. The amount of *Mx1*, *IFI44* and *IFIT1* transcripts from a total of 371 TCGA human tissue specimens was analyzed with regard to etiology. Patients with hepatitis C virus (HCV) infection as a risk factor for development of HCC revealed 2-3-fold higher ISG expression levels than the other groups. Mean values and p-values (Kruskal-Wallis test) are indicated.
